# Early Effects of Alpha-Synuclein Depletion by Pan-Neuronal Inactivation of Encoding Gene on Electroencephalogram Coherence between Different Brain Regions in Mice

**DOI:** 10.3390/biomedicines11123282

**Published:** 2023-12-12

**Authors:** Vasily Vorobyov, Alexander Deev, Olga Morozova, Zoya Oganesyan, Anastasia M. Krayushkina, Tamara A. Ivanova, Kirill Chaprov

**Affiliations:** 1 Institute of Cell Biophysics, Russian Academy of Sciences, 142290 Pushchino, Russia; 2 Institute of Theoretical and Experimental Biophysics, Russian Academy of Sciences, 142290 Pushchino, Russia; aadeev@gmail.com; 3 Institute of Physiologically Active Compounds at Federal Research Center of Problems of Chemical Physics and Medicinal Chemistry, Russian Academy of Sciences, 142432 Chernogolovka, Russia; contact.morozova@gmail.com (O.M.); chapkir@gmail.com (K.C.); 4 Institute of Molecular Medicine, Moscow State Medical University (Sechenov’s University), 119991 Moscow, Russia; philosopher300@yandex.ru; 5 Department of Pharmacology and Clinical Pharmacology, Belgorod State National Research University, 308015 Belgorod, Russia

**Keywords:** tamoxifen, electroencephalogram, coherence, cortex, putamen, ventral tegmental area, substantia nigra, alpha-synuclein

## Abstract

Inactivation of the Snca gene in young mice by chronic injections of tamoxifen (TAM), a selective estrogen receptor modifier, has been shown to decrease the level of alpha-synuclein, a key peptide in the pathogenesis of Parkinson’s disease. In young mice, different time courses of the effect were observed in different brain areas, meaning associated disturbances in the intracerebral relations, namely in brain function after TAM-induced synucleinopathy. Methods: We analyzed electroencephalogram (EEG) coherence (“functional connectivity”) between the cortex (MC), putamen (Pt), and dopamine-producing brain regions (ventral tegmental area, VTA, and substantia nigra, SN) in two groups of two-month-old male mice. We compared EEG coherences in the conditional knockout Snca^flox/flox^ mice with those in their genetic background (C57Bl6J) one, two, and three months after chronic (for five days) intraperitoneal injections of TAM or the vehicle (corn oil). The EEG coherences in the TAM-treated group were compared with those in the alpha-synuclein knockout mice. Results: A significant suppression of EEG coherence in the TAM-treated mice versus the vehicle group was observed in all inter-structural relations, with the exception of MC-VTA at one and three months and VTA-SN at two months after the injections. Suppressive changes in EEG coherence were observed in the alpha-synuclein knockout mice as well; the changes were similar to those in TAM-treated mice three months after treatment. Conclusion: our data demonstrate a combined time-dependent suppressive effect induced by TAM on intracerebral EEG coherence.

## 1. Introduction

The dysfunction of dopaminergic neurons in the substantia nigra (SN) and/or ventral tegmental area (VTA) of the brain is well known to be responsible for symptoms of Parkinson’s disease (PD). The dysfunction has been shown to be associated with the formation of toxic alpha-synuclein aggregates (Lewy bodies/Lewy neurites) in the affected DA cells. This is accompanied by functional alpha-synuclein depletion in the cells [1] that affects synaptic (pre-synaptic, predominantly) mediation, resulting in the dopaminergic system malfunctioning typical in PD [2,3]. Surprisingly, the alpha-synuclein elimination did not affect the neurotransmission of dopamine (DA) [4], hypothetically due to a high neuronal plasticity that compensates for an inherited loss of alpha-synuclein activity by other members of the synucleins family and seemingly associated with the high homology of their amino acid sequences [5]. Alpha-synuclein is well known to bind with neuronal lipid membranes, modifying the neurotransmitter release through the clustering of synaptic vesicles and chaperoning the assembly of SNARE complexes [6]. However, the affinity of alpha-synuclein for synaptic vesicle membranes is reduced by the binding of beta- or gamma-synuclein to alpha-synuclein on the surface of synaptic vesicles [7]. A line of genetically modified mice was developed [8] with conditional pan-neuronal and inactivated Snca gene encoding alpha-synuclein via Cre-recombinase conjugation to the estrogen receptor (ER-T2), which is activated by tamoxifen (TAM). TAM has been shown to downregulate the expression of alpha-synuclein, regardless of the animal’s age (six and twelve months) [9]. However, in eighteen-month-old mice, TAM administration was accompanied by significant changes in dopamine (DA) metabolism, in particular, by a decrease of DOPAC (3,4-dihydroxyphenylacetic acid), a product of oxidative deamination of DA, and HVA (homovanillic acid), its terminal metabolite. In young (two-month-old) conditional knockout Snca^flox/Δflox-NSE/Cre-ERT2^ mice, significant depletion of the alpha-synuclein protein was revealed in various brain areas (the cortex, the striatum, and the midbrain area) in the three months after TAM administration [10]. In parallel with alpha-synuclein depletion, TAM is expected to be able to inhibit the dopamine transporter (DAT) function independent of TAM activity as a selective estrogen receptor modulator [11]. This raises the question about the involvement of the dopaminergic system in the effects initiated by TAM. In addition, TAM has been shown, on one hand, to protect memory and decrease striatal DA metabolism in amyloidosis mice [12], and on the other hand, to impair learning and memory functions in normal mice [13].

It is well known that disturbances in the functioning of neuronal networks in or between different brain structures are able to affect an onset and progression of neurodegenerative diseases [14]. Relative changes in frequency spectra of electroencephalograms (EEGs) [15] recorded from different brain structures and coherence/synchrony of their EEG instantaneous values have been shown to be useful tools [16] for indirect and direct measure, respectively, of “functional connectivity” in normal and diseased brains. Recently, the efficacy of the EEG approaches has been demonstrated in our studies on knockout mice lacking alpha-, beta-, and gamma-synucleins in all possible combinations [17] and on mice models of both Alzheimer’s disease and amyotrophic lateral sclerosis [18]. In the current study, we analyze the early effects of a TAM-initiated conditional pan-neuronal inactivated *Snca* gene and its progression by measuring the levels of baseline EEG coherence between the cortex (MC), putamen (Pt), ventral tegmental area (VTA), and substantia nigra (SN) one, two, and three months after chronic (for five days) injections of TAM or the vehicle (corn oil) in ready-to-use two-month-old mice of a conditional knockout Snca Snca^flox/Δflox-NSE/Cre-ERT2^ line [10]. A combined time-dependent suppressive effect of alpha-synuclein depletion on the intracerebral EEG coherence was shown. In an additional two groups of three–four-month-old alpha-synuclein knockout and control mice, the suppressive changes in EEG coherence were about similar to those observed in the conditional knockout mice three months after TAM injection.

## 2. Materials and Methods

### 2.1. Animals

The generation of mouse lines and the breeding of experimental animals used in this study have been described earlier in [10]. Briefly, all lines were previously established and maintained on a pure C57Bl6J (Charles River) genetic background in our Bioresource Collection of the IPAC RAS Centre for collective use (covered by framework FFSN-2021-0005) and provided for this experiment. Three–four-month-aged alpha synuclein knockout mice, B6(Cg)-Snca^tm1.2Vlb^/J [delta flox KO], maintained on the C57BL/6J genetic background, were studied as well [19].

Up to the age of two months, the animals (all males) were housed in groups of five per cage in a Specific Pathogen Free (SPF) facility, while after TAM injections, each of them was kept in an individual cage. Mice were housed in a standard environment (12/12 h light/dark cycle, 22–25 °C, 30–70% humidity) with food and water ad libitum. The procedures were carried out in accordance with the “Guidelines for accommodation and care of animals. Species-specific provisions for laboratory rodents and rabbits” (GOST 33216-2014), in compliance with the principles enunciated in Directive 2010/63/EU on the protection of animals used for scientific purposes, and approved by the local Institute Ethics Review Committee. Efforts were made to minimize the number of animals and their suffering.

### 2.2. Genotyping

All mice were checked for the presence of the modification in the genome by genotyping using conventional PCR, as described in [10]. Total DNA was isolated from ear biopsies (~30 mg) and used as a template in a PCR reaction with primers:

A_Int1For TGC TGG GCA CAG TGT TGA TTG

A_Int1Rev AAA GGC TGG GCT TCA AGC AG

CRE_rev CAT GAG TAC TTG TGG CTC AC

As a result of amplification, samples of 280 bp and 406 bp fragments were formed from Snca^Δflox/Δflox^ and Snca^flox/flox^ animals, respectively.

### 2.3. Alpha-Synuclein Depletion and Procedures

In the main groups, all the mice were intraperitoneally (i.p.) injected for five consecutive days either with tamoxifen (TAM, CAS # 10540-29-1, Sigma-Aldrich, Darmstadt, Germany) at a dose of 0.5 mmol/kg or with the vehicle (corn oil) in the control group. All animals were randomly divided into two groups: one for EEG and one for molecular analysis.

EEGs were recorded one, two, and three months after TAM/vehicle injections in 6/6, 6/5, and 9/5 mice, respectively, or at the age of three–four-months in alpha-synuclein knockout mice (n = 13) and their littermates (n = 11). The electrodes were implanted 8 days before EEG recording sessions.

In each group, the mice were euthanized via cervical dislocation the day after final EEG registrations; their brains were dissected for further morphological analysis and an evaluation of electrode position. From the left side of the brain, the dorsal striatum was isolated in the cold; dopamine (DA) and its metabolites were measured via HPLC with electrochemical detection, while the prefrontal cortex was extracted for mRNA expression via Real-Time PCR.

### 2.4. Preparation of Histological Sections and Immunohistochemistry

**Figure 1:**
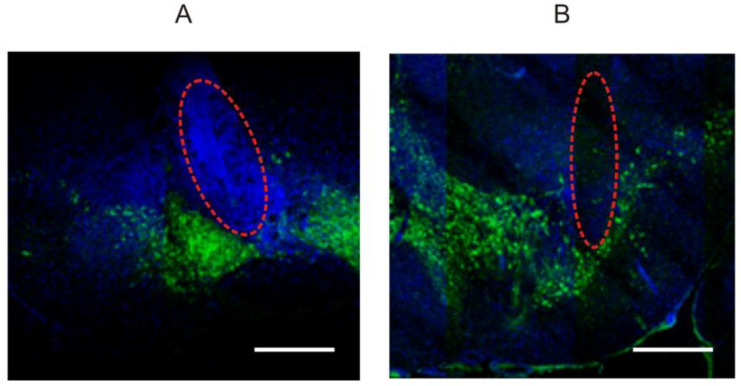
Images of coronal sections of the mouse brain demonstrate coagulated tissues at the position of electrode tips (red dashed ellipses) in the ventral tegmental area (**A**) and substantia nigra (**B**). Dopaminergic neurons (green signal) were immunostained with antibodies against tyrosine hydroxylase, while DAPI-stained nuclei are denoted by blue signals. Scale bar is 500 μm.

After euthanasia, the brain of each mouse was dissected and fixed at 4 °C overnight in Carnoy’s solution (ethanol:chloroform:glacial acetic acid in proportions of 60:30:10, respectively). The brain’s 8-µm thick sections were stained with primary anti-tyrosine hydroxylase antibody (TH, mouse monoclonal antibody, Sigma-Aldrich, Germany, diluted 1:1000) and secondary Goat anti-mouse IgG (H + L) antibodies (Alexa Fluor 488, Thermo A11029 diluted 1:1000), as described in [20]. Briefly, the margins of SN/VTA regions on stained sections were outlined using ZEN black Microscopy Software 2.3 SP1 (Carl Zeiss, Jena, Germany) and an atlas of TH-positive cells [21] (see representative examples in Figure 1).

### 2.5. High-Pressure Liquid Chromatography (HPLC) with Electrochemical Detection

In the brain samples, the levels of serotonin (5-Hydroxytryptamine, 5-HT), dopamine (DA), and its metabolites 3,4-dihydroxyphenylacetic acid (DOPAC) and homovanillic acid (HVA), were evaluated via HPLC with electrochemical detection, as described in [5]. The dorsal striatum tissue was homogenized in 0.06 M HClO_4_ (Sigma Aldrich, Saint Louis, MO, USA) and centrifuged at 15,000× *g* for 15 min at 4 °C. The protein concentrations in the pellets afterward were measured using a Pierce™ BCA Protein Assay kit (Thermo Scientific, Waltham, MA, USA) according to manufacturer’s instructions.

HPLC separation using a liquid chromatograph LC-20ADsp (Shimadzu Corporation, Kyoto, Japan) was carried out on a preliminarily calibrated reversed-phase column C18 Microsorb MVTM (4.6 × 150 mm, Varian, Palo Alto, CA, USA). Detection was performed using a Decade II electrochemical detector (Antec Scientific, Alphen aan den Rijn, The Netherlands), which was equipped with a working glassy carbon electrode 0.85 V and Ag/AgCl reference electrode. To measure the concentrations of DA and its metabolites, a solution containing 0.1 M citrate-phosphate buffer, 0.3 mM 1-octanesulfonic acid sodium salt, and 0.1 mM EDTA (pH 3.1) and 12% methanol, was used as a mobile phase. The monoamine concentrations were calculated using the standard method using a calibration curve.

### 2.6. Analysis of mRNA Expression

The prefrontal cortex samples were snap-frozen in dry ice and kept at −80° C. Total RNA was isolated from tissues with an Extract RNA (Evrogen, Moscow, Russia), according to the chloroform/isopropanol manufacturer’s instructions. Extracted RNA was dissolved in RNase free water and incubated for 5 min at 55° C. Its concentration was determined with a NanoDrop 2000 spectrophotometer (NanoDrop Technologies, Wilmington, DE, USA), and 1500 ng of total RNA was reversely transcribed using the Transcriptor First Strand cDNA Synthesis Kit (MMLV RT kit, Eurogen, Russia). For PCR amplification reaction, the buffer of SYBR Green I intercalating dye and Hot Start polymerase 5X qPCR mix-HS SYBR (PK147L, Evrogen, Russia) was used. Each sample was analyzed in triplicate on a 384-well QuantStudio™ 7 Flex System (Applied Biosystems, Göteborg, Sweden) while the obtained data were evaluated using integrated QuantStudio™ Real-Time PCR Software v 1.3 (Applied Biosystems, Darmstadt, Germany). An amount of cDNA for each gene was normalized to that of GAPDH in each group of mice. Primer sequences used were as follows:

GAPDH_ rev GAG CTT CCC GTT CAG CTC TG

GAPDH_ for ATG ACC ACA GTC CAT GCC ATC

Snca_rev TGA ACA CAT CCA TGG CTA AAG A

Snca_for CTG CCC TTG CCT CTT TCA TTG

Sncb_rev ATG CCT GCT CCT TGG TTT TCT

Sncb_for CAA GGA AGG CGT CCT CTA TGT

SNCG_rev CAA CAC AGT GGC CAA CAA GA

SNCG_for GGG GTT CCA AGT CCT CCT T

### 2.7. Implantation of Electrodes

Each mouse was subcutaneously injected with tiletamine/zolazepam (25 mg/kg, Zoletil^®^, Virbac, Carros, France) and xylazine (2.5 mg/kg, Rometar^®^, Bioveta, Ivanovice na Hané, Czech Republic) mixture. EEG recording electrodes were implanted into the left motor cortex and putamen (MC and Pt; AP: +1.1 mm anterior to bregma; ML: ±1.5 mm lateral to midline; DV: −0.75 and −2.75 mm below the skull surface, respectively), into the left ventral tegmental area (VTA; AP: −3.1, ML: −0.4, DV: −4.5) and the right substantia nigra (SN; AP: −3.2, ML: +1.3, DV: −4.3) [22]. The electrodes were prepared from two varnish-insulated nichrom wires (100 µm in diameter) glued together with tips free from insulation for 100 µm. The reference and ground electrodes (stainless steel wire, 0.4 mm in diameter) were positioned over the caudal part of the brain (AP: −5.3, ML: ±1.8, DV: −0.5). A computerized 3D stereotaxic StereoDrive (Neurostar, Tübingen, Germany) was used for precise positioning of all electrodes. The electrodes were fixed to the skull with dental cement and soldered to a mini-connector (Sullins Connector Solutions, San Marcos, CA, USA).

### 2.8. Baseline EEG Recording and Computation of the EEG Coherence

In the three days after the implantation, each mouse was daily placed in an electrically shielded experimental cage and connected to a cable plugged into a digital Neuro-MEP amplifier (Neurosoft Ltd., Ivanovo, Russia). On day 8, a 30-min baseline EEG was recorded after a 20–30-min adaptation to the environment. EEG signals were amplified, filtered (0.1–35 Hz), and sampled (1 kHz) on-line by the amplifier and kept in memory of an operational computer for further analysis. The program allowed both automatic and manual rejection of EEG fragments containing artifacts and epileptic spikes. EEG data were processed using custom prepared software “EEG Tools” (6.7.0 version) based on Borland C++ Builder 6.0. It was developed by A.D. and supported by the Government Contract No 075-01025-23-01 with the Institute of Theoretical and Experimental Biophysics, Russian Academy of Sciences. EEG coherence was evaluated in the range of 1–30 Hz, with the averaging of data in 1-Hz bins and in “classical” EEG bands (in Hz): delta 1 (1–2), delta 2 (2–4), theta (4.0–8.0), alpha (8–12 Hz), beta 1 (12–20), and beta 2 (20–30.0). The values of coherence in the bands were averaged for every successive 10-min interval (for further statistical analysis) and totally for 30 min (for illustrations). (See details in [18]).

### 2.9. Statistics

Differences in EEG coherencies, averaged for 10 min in frames of individual frequency bands or the whole spectra, in both sets of TAM- versus vehicle-injected mice, and in alpha-synuclein knockout versus non-transgenic mice, were analyzed via 2-way ANOVA for repeated measures using STATISTICA 10 (StatSoft, Inc., Tulsa, OK, USA). For multiple comparisons, the Bonferroni post hoc test was applied. Results were considered statistically significant at *p* < 0.05. All data are shown as mean ± SEM.

## 3. Results

**Figure 2:**
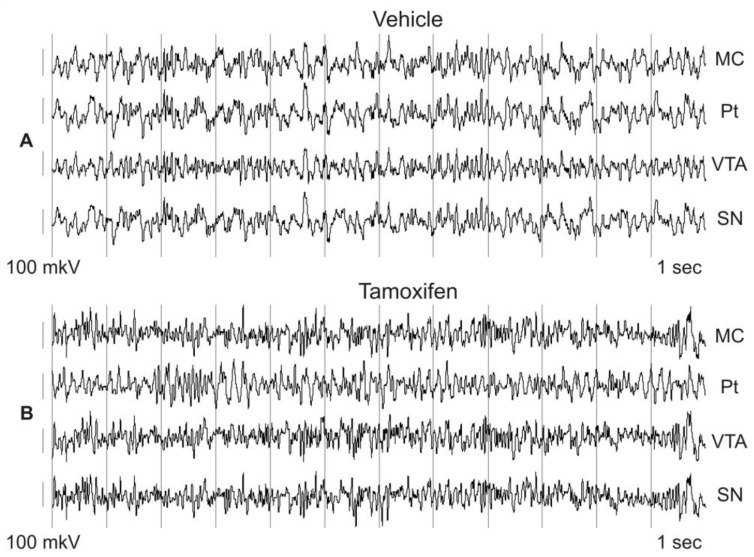
Representative patterns in 12 s fragments of baseline EEG in wakeful and behaviorally active 3-month-old mice intraperitoneally injected at the age of 2 months with the vehicle (corn oil, (**A**)) or tamoxifen (0.5 mmol/kg, (**B**)). EEGs were recorded from the motor cortex (MC), putamen (Pt), ventral tegmental area (VTA), and substantia nigra (SN). Time calibration is 1 s, amplitude calibration is 100 mkV.

In the raw EEGs, evident differences in the intracerebral synchronization were observed between tamoxifen (TAM)- and vehicle-treated mice (Figure 2).

**Figure 3:**
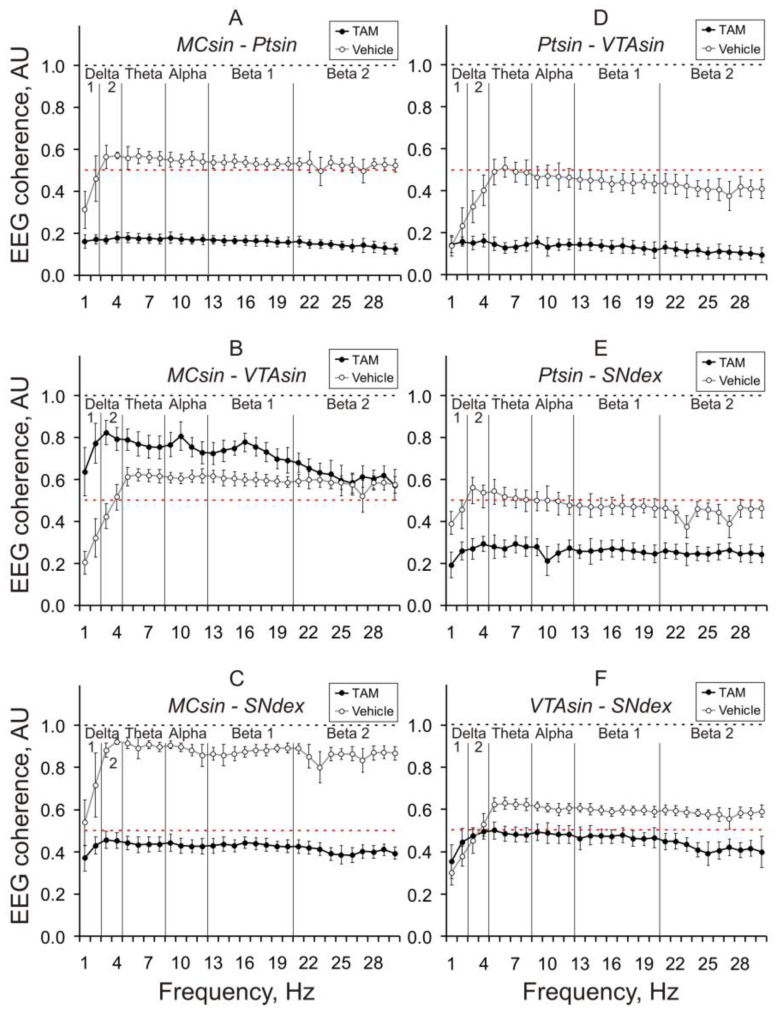
Inter-structural baseline EEG coherences in 3-month-old mice that were averaged for 10-min intervals one month after intraperitoneal injection of tamoxifen (TAM, 0.5 mmol/kg) or the vehicle (black and grey lines, respectively). MC, Pt, VTA, and SN are the motor cortex, putamen, ventral tegmental area, and substantia nigra (SN), respectively. Inter-structural coherence is denoted on the plates as MC-Pt (**A**); MC-VTA (**B**); MC-SN (**C**); Pt-VTA (**D**); Pt-SN (**E**), and VTA-SN (**F**). Ordinate is the average value of EEG coherence in each of the 1-hertz (Hz) bins within the analyzed 30-Hz frequency range denoted on abscissa. Five vertical lines separate “classical” EEG frequency bands (from left to right: delta 1, delta 2, theta, alpha, beta 1, and beta 2, respectively). Black and red dashed lines demonstrate maximal (1.0) and middle (0.5) coherence values, respectively.

This was readily visible after averaging of the inter-structural coherences for consecutive 10-min intervals. In Figure 3, coherence averaging is presented in 10–20-min intervals after the injections. TAM vs. control mice were characterized by significant suppression of EEG coherence between different brain areas practically in all frequency bands. The only exception was associated with enhanced EEG coherence between MC and VTA in control vs. TAM mice (Figure 3B).

**Figure 4:**
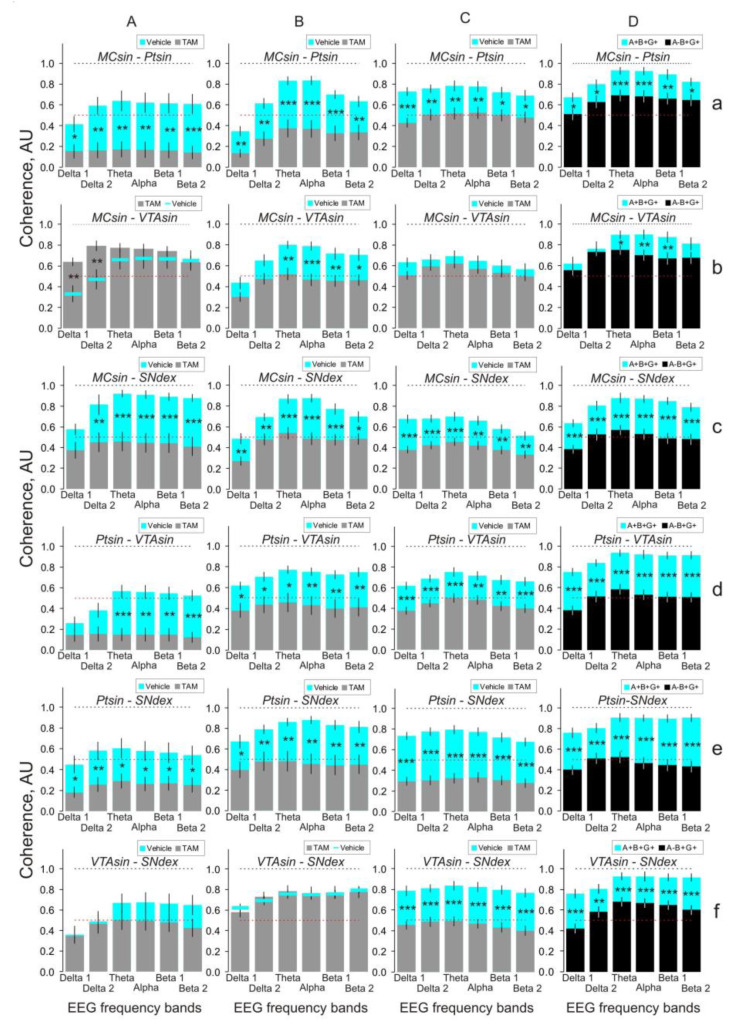
Inter-structural baseline EEG coherences that were averaged for 30 min 1, 2, and 3 months ((**A**), (**B**), and (**C**), respectively) after injection of tamoxifen (TAM, 0.5 mmol/kg) or the vehicle (grey and blue bars, respectively) in comparison with those in control and alpha-synuclein knockout mice ((**D**), blue and black bars, respectively). MC, Pt, VTA, and SN are the motor cortex, putamen, ventral tegmental area, and substantia nigra (SN), respectively. Inter-structural coherence is denoted on the plates as MC-Pt (**a**); MC-VTA (**b**); MC-SN (**c**); Pt-VTA (**d**); Pt-SN (**e**), and VTA-SN (**f**). Ordinate is the average value of EEG coherence in “classical” EEG frequency bands denoted on abscissa (from left to right: delta 1, delta 2, theta, alpha, beta 1, and beta 2, respectively). Black and red dashed lines demonstrate maximal (1.0) and middle (0.5) coherence values, respectively. Star symbols denote significant differences of coherence in EEG frequency bands between TAM- and vehicle-treated mice (**A**–**C**) and between alpha-synuclein knockout and control mice (**D**), where *, **, and *** denote *p* < 0.05, *p* < 0.01, and *p* < 0.001, respectively. (The results of two-way ANOVA analysis of coherences in different frequency bands are seen in Appendix A).

The effects were stable in consecutive 10-min intervals and allowed the averaging of the data for the full (30-min) period of EEG recordings (Figure 4).

In spite of an overall suppression of the EEG coherence in TAM versus vehicle mice, several interrelations (MC-VTA in one- and three-month groups, and VTA-SN in one- and two-month groups, see Figure 4A,C and Figure 4A,B, respectively) were practically insensitive to TAM (see Appendix B). The spectral profiles of intracerebral EEG coherent interrelations in in the alpha-synuclein knockout versus control mice roughly coincide with those observed in TAM- versus vehicle-treated mice three months after the injections (c.f., Figure 4D and Figure 4C, respectively).

**Figure 5:**
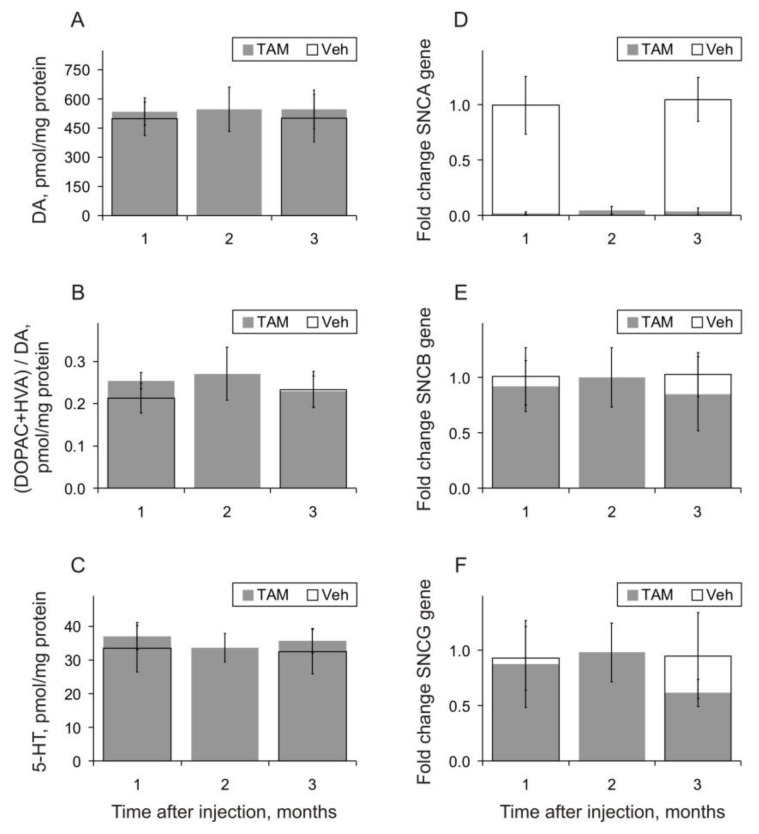
Dorsal striatum concentrations of (**A**) dopamine (DA), (**B**) its metabolites of 3,4-dihydroxyphenylacetic acid (DOPAC) and homovanillic acid (HVA) presented as their relative ratios, and (**C**) 5-Hydroxytryptamine (5-HT, serotonin) one, two, and three months (abscissa) after TAM (grey bars) and one and three months after vehicle (open bars) injections. The normalized expression levels of mRNA for alpha-, beta-, and gamma- synucleins in the prefrontal cortex in different intervals after TAM injections are presented on (**D**), (**E**), and (**F**) plates, respectively. GAPDH gene expression was used as a reference value for normalization.

DA and its metabolite levels were quantified using HPLC in the dorsal striatum, where dopaminergic synapses from SN neurons are remarkable. After TAM application, no significant changes in levels of DA and its metabolites were observed in any of the groups (Figure 5A,B, grey bars). In mice from both (one and three month) control groups, these parameters were practically identical.

The levels of 5-HT, measured in the same brain samples via HPLC, were relatively stable after TAM application (in pmol/μg protein): 33.43 ± 6.881, 37.10 ± 4.057, and 33.66 ± 4.211 at one, two, and three months, respectively (Figure 5D, grey bars). In mice from the control groups, the values of 5-HT concentrations were practically the same: 35.68 ± 3.637 and 32.47 ± 6.644 pmol/μg protein at one and three months after the vehicle injection, respectively (Figure 5D, open bars). Analysis of mRNA for alpha-synuclein revealed a significant reduction in expression levels in the prefrontal cortex following TAM injections (Figure 5D, grey bars). In contrast, no significant differences in the levels of beta- or gamma-synuclein expressions in the same brain regions following TAM-induced inactivation of the Snca gene were observed (Figure 5E,F, grey bars).

## 4. Discussion

In this study on mice, we demonstrated a dominated suppression (with a few exceptions) of EEG coherence/synchronization in the interrelations between different brain structures at one, two, and three months after tamoxifen (TAM) versus vehicle injections. The three-month delayed effects initiated by TAM were about similar to those observed in the alpha-synuclein knockout mice. Together, these allow making three preliminary conclusions: (a) TAM triggers mechanisms of specific suppressive interrelations between different brain areas; (b) the phasic evolution of TAM-initiated effects supposedly indicates more than one mechanism involved in these effects; and (c) delayed effects initiated by TAM might be associated, at least in part, with an inactivation of alpha-synuclein in the brain.

Indeed, the latter is in line with the results of our previous study where three months after identical TAM administration, significant depletion of alpha-synuclein levels in different brain regions (the cortex, striatum, and midbrain) was revealed [10]. Furthermore, the similarities between corresponding EEG coherent interrelations between “TAM/vehicle” and “alpha-synuclein knockout/control” groups (c.f., Figure 4c,d) confirm indirectly an association of these interrelations with elimination/deactivation of the brain’s alpha-synuclein. Interestingly, EEG coherence evolution in the “TAM” group was evidently specific in interrelations between different brain areas. The MC-Pt and Pt-VTA coherences increased from 0.15 one month after the TAM injection up to 0.4–0.5 two months later, approximately reaching those in the knockout mice (see grey and black bars on Figure 4a,d). The TAM-initiated effect on the MC-VTA synchronization (grey bars on Figure 4b) was paradoxical and characterized by a relatively high (0.7–0.8) level of coherence one month (A) after TAM injection (similar to that in the knockouts, D) and by lesser levels of EEG synchronization at two and three months (B and C, respectively). Finally, TAM initiated a unique delayed effect on the VTA-SN coherence with a significant increase (up to that in the vehicle group) at two months, and attenuating three months after the injections (see grey bars on Figure 4f). Inter-structural synchronization is well known to be closely linked with memory processes [23]. On the other hand, TAM has been shown to affect many brain structures and functions [24] seemingly in part via TAM-induced neuronal stress evoked by inhibiting the cholesterol synthesis [25]. As revealed in our previous study, time-dependent TAM-associated “mosaic” effects on EEG coherences between the cortex and other brain structures (Figure 4) partly allow the explanation of the contradictive effects of TAM on cognitive functions [12,13,14,15,16,17,18,19,20,21,22,23,24,25] and the duality of its neuropsychiatric action [26]. On the other hand, the “mosaic” TAM-initiated effects on the coherence interrelations between the brains regions might be linked with the complicity of the steroid-dopamine interaction mechanisms in the brain (for review, see [27]). In particular, TAM may directly suppress the DA reuptake transporter without involvement of estrogen receptors [11,28] and has been shown to exert a weak competitive inhibition of the DA antagonist site of DA_2_ receptors in the striatum [29]. In addition, TAM has been found to alter DA output via direct, non-genomic effects on the nigrostriatal dopaminergic system in the brain [30]. Thus, abnormally changed levels of DA in the extracellular space provoked by TAM administration are expected and may disrupt natively normal EEG coherence/synchronization between different brains regions observed in the control group (c.f., grey and blue bars in Figure 4). These should be taken into account in an analysis of the mechanisms of alterations in social interaction, locomotor activity, and anxiety that were revealed in TAM-treated mice [31]. As mentioned above, TAM-produced EEG coherence increasing between MC and Pt in periods from one to three months after its administration (see gray bars on Figure 4A–C,a) coincided with a striatal alpha-synuclein level drop that was revealed in our previous experiments [10]. The specific role of the striatum (putamen) in TAM-induced effects is highlighted by a relatively high and stable EEG coherence between VTA and SN for two months after TAM injection (see gray bars on Figure 4A,B,f), whereas the level of alpha-synuclein in the midbrain tended to be diminished [10]. On the other hand, three months after TAM administration, when the levels of alpha-synuclein in the midbrain were negligible, VTA-SN coherence was robustly diminished as well (Figure 4C,f).

In our study, TAM application was ineffective in the initiation of dopaminergic system activity changes, which was evident in stable levels of both DA and its metabolites (see Figure 5A and Figure 5B, respectively). These suggest that the amount of DA used at the synaptic terminals of dopaminergic neurons exceeded that of newly synthesized DA, meaning that TAM-produced induction did not exert any detrimental effects on DA status in the dorsal striatum of the alpha-synuclein conditional mice. Taking in to account that this was accompanied by the inhibiting effects of TAM on alpha-synuclein expression (see Figure 5D) and the level of this protein [10], these allow for the conclusion that this drug has no detrimental influence on dopaminergic activity sourced from SN. At first approximation, the same seems to be characteristic for the serotoninergic system (see Figure 5C). The lack of increased expression of beta- and gamma-synuclein homologues (see Figure 5E and Figure 5F, respectively) suggests that the amount of these proteins accumulated in the brain is sufficient for autonomous synaptic stabilization despite the alpha-synuclein dysfunction, which is in line with interrelations revealed between the synucleins [7]. In contrast to our results obtained on relatively young mice, the TAM-induced dysfunctional effect on the alpha-synuclein in aging animals has been shown to be accompanied by perceptible functional changes in the nigrostriatal system [9]. This might exacerbate PD pathology development; thus, when aiming to decrease the possible adverse effects of TAM treatment, it should be started at the early stages of the disease.

## 5. Conclusions

For three months after the intraperitoneal administration of a selective estrogen receptor modulator, tamoxifen, targeted to decrease the level of alpha-synuclein, significant alterations in the synchronization of different brain area functions were observed. The measurements of intra-cerebral EEG coherence allow the detailed analysis of the involvement of different mechanisms in the early effects initiated by tamoxifen. Slow depletion of alpha-synuclein protein levels in the brain is hypothesized to be one of these mechanisms. Together, the data obtained highlight possible adverse effects of the therapy, directed to reduce normal physiological alpha-synuclein levels, on patient’s mood and cognition in clinic.

## Data Availability

Data is contained within the current article.

## Appendix A

**Table A1:** Two-way ANOVA analysis of EEG coherence in separate frequency bands between various brain areas (A–F) in mice in one, two, and three months after tamoxifen administration and in alpha-synuclein knockout mice.

**Brain Areas**	**MCsin—Ptsin (A)**							
**Drugs/Mice**	**Tamoxifen (TAM) vs. vehicle**						**Knockouts Versus Normal Mice**	
**Months after TAM**	**1**		**2**		**3**			
**Bands/Coherence**	**F_30_**	***p***	**F_27_**	***p***	**F_36_**	***p***	**F_66_**	***p***
delta1	5.8	0.022	10.6	0.003	18.7	<0.001	4.8	0.032
delta2	12.9	0.001	12.8	0.001	10.7	0.002	4.4	0.039
theta	12.5	0.001	19.1	<0.001	9.9	0.002	13.1	<0.001
alpha	11.8	0.002	19.8	<0.001	8.6	0.005	12.0	<0.001
beta1	11.9	0.002	14.6	<0.001	6.7	0.012	10.3	0.002
beta2	14.1	<0.001	8.6	0.007	6.4	0.014	5.5	0.022
**Brain Areas**	**MCsin—VTAsin (B)**							
**Drugs/Mice**	**Tamoxifen (TAM) vs. vehicle**						**Knockouts Versus Normal Mice**	
**Months after TAM**	**1**		**2**		**3**			
**Bands/Coherence**	**F_30_**	***p***	**F_27_**	***p***	**F_36_**	***p***	**F_66_**	***p***
delta1	10.1	0.003	3.1	0.091	2.8	0.052	1.2	0.276
delta2	9.1	0.005	4.1	0.053	1.9	0.168	0.3	0.616
theta	1.1	0.298	12.5	0.002	2.4	0.130	5.6	0.041
alpha	0.7	0.422	15.4	<0.001	2.4	0.128	10.0	0.002
beta1	0.4	0.525	9.5	0.005	1.9	0.173	9.5	0.005
beta2	0.1	0.897	7.6	0.010	1.8	0.191	4.3	0.054
**Brain Areas**	**MCsin—SNdex (C)**							
**Drugs/Mice**	**Tamoxifen (TAM) vs. vehicle**						**Knockouts Versus Normal Mice**	
**Months after TAM**	**1**		**2**		**3**			
**Bands/Coherence**	**F_30_**	***p***	**F_27_**	***p***	**F_36_**	***p***	**F_66_**	***p***
delta1	3.7	0.065	9.2	0.005	22.5	<0.001	17.4	<0.001
delta2	11.5	0.002	7.8	0.009	15.4	<0.001	15.8	<0.001
theta	20.8	<0.001	21.2	<0.001	12.2	<0.001	23.6	<0.001
alpha	20.5	<0.001	29.1	<0.001	11.1	0.001	24.7	<0.001
beta1	18.9	<0.001	16.4	<0.001	8.5	0.005	26.6	<0.001
beta2	22.2	<0.001	6.8	0.015	7.6	0.008	18.3	<0.001
**Brain Areas**	**Ptsin—VTAsin (D)**							
**Drugs/Mice**	**Tamoxifen (TAM) vs. vehicle**						**Knockouts Versus Normal Mice**	
**Months after TAM**	**1**		**2**		**3**			
**Bands/Coherence**	**F_30_**	***p***	**F_27_**	***p***	**F_36_**	***p***	**F_66_**	***p***
delta1	1.2	0.291	6.6	0.016	16.5	<0.001	43.4	<0.001
delta2	3.7	0.636	7.1	0.013	12.6	<0.001	25.3	<0.001
theta	14.9	<0.001	7.3	0.012	12.0	<0.001	33.1	<0.001
alpha	12.5	0.001	8.2	0.008	11.1	0.001	38.0	<0.001
beta1	12.0	0.002	8.7	0.007	11.4	0.001	37.2	<0.001
beta2	13.8	<0.001	9.1	0.005	12.5	<0.001	36.3	<0.001
**Brain Areas**	**Ptsin—SNdex (E)**							
**Drugs/Mice**	**Tamoxifen (TAM) vs. vehicle**						**Knockouts Versus Normal Mice**	
**Months after TAM**	**1**		**2**		**3**			
**Bands/Coherence**	**F_30_**	***p***	**F_27_**	***p***	**F_36_**	***p***	**F_66_**	***p***
delta1	6.6	0.015	7.4	0.011	46.2	<0.001	25.2	<0.001
delta2	7.7	0.009	7.9	0.009	40.5	<0.001	14.8	<0.001
theta	5.8	0.022	10.7	0.003	35.4	<0.001	34.2	<0.001
alpha	6.4	0.017	13.1	0.001	31.4	<0.001	37.0	<0.001
beta1	5.3	0.029	11.3	0.002	28.7	<0.001	37.7	<0.001
beta2	5.2	0.029	10.1	0.004	32.1	<0.001	41.9	<0.001
**Brain Areas**	**VTAsin—SNdex (F)**							
**Drugs/Mice**	**Tamoxifen (TAM) vs. vehicle**						**Alpha-Knockouts vs. Normal Mice**	
**Months after TAM**	**1**		**2**		**3**			
**Bands/Coherence**	**F_30_**	***p***	**F_27_**	***p***	**F_36_**	***p***	**F_66_**	***p***
delta1	0.0	0.916	0.5	0.501	34.6	<0.001	24.7	<0.001
delta2	0.0	0.874	0.3	0.612	26.3	<0.001	8.8	0.004
theta	1.3	0.257	0.1	0.760	27.2	<0.001	18.9	<0.001
alpha	1.5	0.236	0.0	0.869	28.7	<0.001	18.2	<0.001
beta1	1.6	0.215	0.0	0.871	31.2	<0.001	37.7	<0.001
beta2	2.45	0.128	0.0	0.833	30.3	<0.001	22.6	<0.001

## Appendix B

**Table A2:** Two-way ANOVA analysis of the EEG coherence spectra between various brain areas in mice in one (A), two (B), and three (C) months after tamoxifen (TAM) administration and in alpha-synuclein knockout mice.

Drugs/Mice	Tamoxifen (TAM) vs. vehicle						Knockouts versus Normal Mice (D)	
Months after TAM	1 (A)		2 (B)		3 (C)			
Brain Areas	F_180_	*p*	F_162_	*p*	F_216_	*p*	F_396_	*p*
MCsin—Ptsin	68.4	<0.001	83.7	<0.001	23.3	<0.001	46.4	<0.001
MCsin—VTAsin	19.8	0.001	48.7	<0.001	3.7	0.055	8.8	0.032
MCsin—SNdex	90.9	<0.001	82.6	<0.001	15.5	<0.001	125	<0.001
Ptsin—VTAsin	51.7	<0.001	46.9	<0.001	24.4	<0.001	210	<0.001
Ptsin—SNdex	36.5	<0.001	60.3	<0.001	72.2	<0.001	185	<0.001
VTAsin—SNdex	5.2	0.024	0.1	0.864	90.8	<0.001	107	<0.001
